# Age-related variations of the hemodynamic response function spatially resolved across human cerebral cortex

**DOI:** 10.3389/fnagi.2026.1774543

**Published:** 2026-02-25

**Authors:** Nooshin J. Fesharaki, Amanda Taylor, David Ress

**Affiliations:** 1 Neurosurgery Department, University of Texas Health Science Center at Houston, Houston, TX, United States; 2 Physics and Engineering Department, University of St. Thomas, Houston, TX, United States; 3 Department of Neuroscience, Baylor College of Medicine, Houston, TX, United States

**Keywords:** aging, cerebral blood flow, cerebral cortex, functional magnetic resonance imaging, hemodynamic response function

## Abstract

Normal aging leads to regional vascular and neural alterations. Age-related impairments in neurovascular coupling (NVC) affect the blood-oxygen-level-dependent (BOLD) hemodynamic response function (HRF) measured with functional magnetic resonance imaging, causing changes in both amplitude and temporal dynamics. Previously, global, systematic age-related changes in HRF characteristics were demonstrated, consistent with known microvascular aging effects. In this follow-up study, a standard space was used to compare spatially resolved cortical HRF dynamics between sex-balanced groups of young and older adults. The results showed substantial age-related differences in both HRF amplitude and timing across distinct cortical regions. Nearly twice as much cortical area exhibited age-related alterations in amplitude compared with timing parameters, although the spatial patterns of these effects partially overlapped. Regional HRF changes aligned with known spatial patterns of vascular aging. Overall, the findings indicate that normal aging strongly affects NVC, particularly in areas supplied by major cerebral arteries and their watershed regions.

## 1 Introduction

Non-invasive, blood-oxygen-level-dependent (BOLD) functional magnetic resonance imaging (fMRI) measures neural activity indirectly through neurovascular coupling (NVC), linking hemodynamic responses to neurons’ metabolic and oxygen demands (Ogawa et al., 1992).

The hemodynamic response function (HRF), reflecting responses to brief (<4 s) neural activity, underlies BOLD analysis, and typically unfolds in three phases: (1) initial peak, (2) post-stimulus undershoot, and (3) gradual return to baseline (Lindquist et al., 2009).

The interactions of CBF and CMRO_2_ during brief neural activity – presumably via proximal integration signaling – shape the HRF (Kim and Ress, 2016; Drew, 2019). Early models suggested that CBF drives the initial peak, while its proceeding dip and undershoot were attributed to CMRO_2_. The gradual return may also reflect lingering CBF (Kim and Ress, 2016; Kim et al., 2013; Ress et al., 2009).

Normal aging can disrupt NVC, leading to alterations in HRF dynamics (Mueller-Bierl et al., 2007). The disruption may arise from age-related vascular changes, such as reduced arteriolar/capillary perfusion or density (vascular rarefaction) (Bennett et al., 2024), altered vascular branching and vessel length, and decreased pericyte coverage, all of which could lead to a sparser blood distribution network (Sweeney et al., 2016). Endothelial dysfunction further contributes to vascular rarefaction, diminishing microvascular capacity to adequately supply active neural networks. Such hypoperfusion, especially due to upstream arterial damage, disproportionally affects watershed regions, which are located at the border zones between major cerebral arterial territories (Momjian-Mayor and Baron, 2005; Sorond and Lipsitz, 2011; Wang et al., 2018).

At the arteriolar level, aging can promote smooth muscle cell proliferation and migration, which results in vessel wall thickening, reduced elasticity, and consequently impaired vasomotor function (Donato et al., 2018; Yabluchanskiy et al., 2021; Zimmerman et al., 2021). Increased vascular tortuosity with age further alters blood flow dynamics (Bennett et al., 2024; Zimmerman et al., 2021). Moreover, increased large artery stiffness with aging impairs CBF regulation and transmits damaging pulsations to microvessels, exacerbating damage to the microvascular structures and leading to cognitive impairment (Springo et al., 2015; Winder et al., 2021; Jennings et al., 2021).

Aging causes region-specific vascular changes. Hypoperfusion from major cerebral artery damage (e.g., atherosclerosis) increases infarction risk in watershed areas adjoining their territories (Momjian-Mayor and Baron, 2005). Aging also alters the biomechanical properties of the posterior cerebral artery (PCA) and parenchyma arteries (PA) branching from the middle cerebral artery (MCA) (Diaz-Otero et al., 2016). The PCA may be more vulnerable to aging than the anterior cerebral artery (ACA) (Roth et al., 2017), while CBF decline is often greater in the MCA than in the PCA (Flück et al., 2014).

These vascular alterations align with observed regional CBF reductions, where frontal and temporal lobes show greater declines in perfusion and cerebrovascular reactivity (CVR) (Hu et al., 2025; Peng et al., 2018). However, age-related associations between regional brain function and CBF remain inconsistent (Graff et al., 2023). Region-specific findings on CMRO_2_ are similarly mixed: while global increases may compensate for reduced CBF (Peng et al., 2014), regional reductions have been noted (De Vis et al., 2015). Together, these shifts in CBF and CMRO_2_ influence HRF dynamics, complicating BOLD fMRI interpretation.

Age-related changes in the BOLD HRF likely reflect alterations in NVC, which manifest in both amplitude and timing. However, findings on age-related HRF changes have been inconsistent. Some studies have reported reduced HRF amplitude and delayed responses in older adults, particularly in the occipital lobe, though these effects varied by region and often diminish after accounting for gray-matter atrophy (West et al., 2019; Liu et al., 2017). In one eloquent example, McDonough et al. (2025) reported an association between aging and reduced HRF peak amplitude but observed no age-related differences in HRF latency or width (McDonough et al., 2025). Other studies showed no significant differences in HRF dynamics across age groups (D’Esposito et al., 1999; Buckner et al., 2000; Huettel et al., 2001; Brodtmann et al., 2003; Ward et al., 2008; Aizenstein et al., 2004). These contradictory results highlight the need to better characterize age-related changes in HRF dynamics.

Previously, we investigated how aging globally impacts HRF dynamics in adults aged 22–75 (Fesharaki et al., 2024). We estimated the HRF without imposing *a priori* assumptions about its shape and examined age-related changes in its parameters’ spatial means and variabilities. With age, HRF peak amplitude and contrast-to-noise ratio (CNR) became less spatially variable, while the full-width at half-maximum (FWHM) of the HRF became more variable. Additionally, HRF amplitudes were “tuned” to a particular FWHM value, which shifted faster with broader bandwidth as age increased. Aging also increased fast HRF dynamics that we characterized as high-frequency power fraction (HFPF).

Our earlier findings provided evidence of systematic, age-related changes in NVC, aligning with known microvascular aging effects. However, that study lacked sex balance and only analyzed the age effects globally in each subject’s native space. In this follow-up, we compared cortical HRF dynamics between sex-balanced young and older adults, focusing on spatially resolved differences in a common space. We hypothesized that age-related, regional HRF changes would correspond to known spatial patterns of vascular aging.

## 2 Materials and methods

### 2.1 Participants

We collected data from a cohort of 30 healthy right-handed participants with no history of neurological disease during 53 scanning sessions. The study included two sex-balanced age groups: (1) 16 young adults aged 22–30 years (mean age: 25.5 years), with a total of 29 scanning sessions; and (2) 14 older adults aged 53.5–75 years (mean age: 66 years), with a total of 24 scanning sessions. For older individuals, we performed a Mini Mental State Assessment and recruited only those with score ≥ 24. This data was part of our previous published study of the global impact of aging on HRF characteristics (Fesharaki et al., 2024). In the present study, we aimed to maintain a uniform sex distribution within each age group and to have better control for other physiological factors, such as variations in resting CBF before and after menopause (Liu et al., 2016), that may influence HRF measurements. A written, informed consent form was collected for each subject at the time of first enrollment in this study. All participants were screened for MRI safety before each scanning session. We performed all experimental procedures in accordance with a protocol reviewed and approved by the Baylor College of Medicine (BCM) Institutional Review Board and based on the principles of the Belmont Report (Department of Health, Education, and Welfare, National Commission for the Protection of Human Subjects of Biomedical and Behavioral Research, 2014). Each subject was trained on task prior to data collection, and their performance was validated.

### 2.2 Magnetic resonance imaging data collection protocols

Data was collected using a 3T MAGNETOM Prisma Siemens MRI scanner (Siemens Healthcare, Erlangen, Germany) located at BCM and equipped with a 32-channel radio frequency head coil. During each scanning session, we obtained high-resolution T1-weighted anatomical reference volume for each subject using an MP-RAGE sequence: 0.8-mm isotropic voxels, TR = 2,300 ms, T1 = 900 ms, flip angle = 9°, 2 repetitions. Additionally, five T2*-weighted functional scans for each subject were performed using a simultaneous multi-slice (SMS) accelerated echo-planar imaging (EPI) sequence (Breuer et al., 2005): 2-mm isotropic voxels, TR = 1,250 ms, TE = 30 ms, GRAPPA = 2, slice thickness = 2 mm, 60 quasi slices. During each functional scan, the timing of the stimulus was aligned with the TR of the scanner, resulting in 24 TRs per HRF. After every two functional scans, high-order shimming was also performed to reduce off-resonance distortion.

During each session, we also collected a T1-weighted volume before and after performing the functional scans that used the same slice prescription as the functional scans. This scan, referred to as the “inplane” anatomy, was acquired using a 3D FLASH sequence with the minimum TR and TE, flip angle = 15°, field-of-view = 256, slice thickness = 2 mm, 1-mm inplane pixel size, 64 slices). The inplane anatomy was later used to register the functional data to the high-resolution anatomical volume.

### 2.3 Speeded audio-visual-motor sequence-following task (SAST)

**Figure 1:**
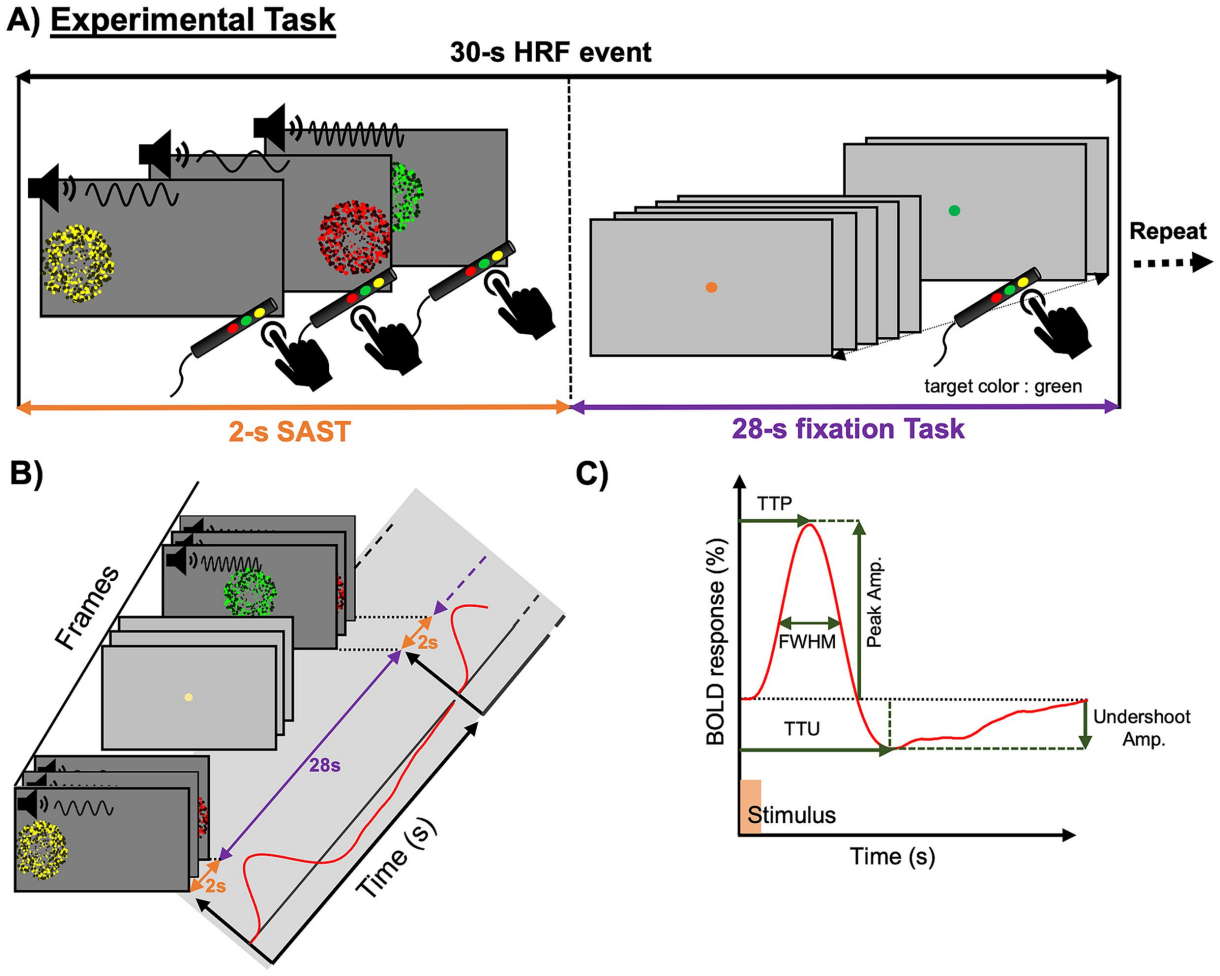
**(A)** An event-related paradigm including epochs of a 2 s speeded audio-visual-motor sequence-following task (SAST) followed by a 28 s non-demanding fixation task. **(B)** The hemodynamic response function (HRF) time-series arises from the SAST with a return to the baseline during the following fixation task. **(C)** Characteristics of the HRF in terms of amplitude and timing parameters.

For each subject, HRFs were evoked using a slow event-related-design experiment, including epochs of a 2-s SAST followed by a 28-s non-demanding, color-detection fixation task (Figures 1A,B). Each HRF event began with a 0.5-s cue, a white-on-black color dot, signaling participants to expect the stimulus on the screen, followed by a 2-s SAST period. The SAST had three components: visual, audio, and response (motor). Visual stimulation consisted of three consecutive presentations of flickering (6 Hz) colored-dot patches. Each patch could be presented in one of three circular (5° diameter) regions uniformly and horizontally distributed across the length of the display for 667 ms. Each position had a specific color: yellow on the left, green in the center, and red on the right. The spatial order of presentation of each patch was random, without sequential repetition. Each patch was paired with a distinct audio stimulus of bandpass-filtered white noise: yellow with medium pitch, red with low pitch, and green with high pitch (Figure 1A, left panel). While following each colored-dot patch with eye movements, subjects were instructed to respond quickly in the order corresponding to the color of the patch using hand-held buttons spatially corresponding to each patch position.

### 2.4 Fixation task

To retain a low-level of attention between the SAST stimuli, subjects were asked to perform a non-demanding, color-detection fixation task for 28 s following each stimulus. During this period, subjects were instructed to maintain fixation on a central dot (0.15° diameter) with a color changing every 0.6 s, and press a button upon the appearance of a single target color (e.g., green), which appeared at random times (exponential distribution) averaging every 6 s.

During each functional scan, there were 16 epochs of a SAST together with a fixation task. With five functional scans during each session, we therefore collected a total of 80 (16 × 5) HRFs per session. Both tasks were designed using MATLAB (the MathWorks, Inc.) with PsychToobox-3 and presented on a 32″ MR-compatible screen (BOLD screen, Cambridge Research Systems, Kent, United Kingdom) mounted at the back of the MRI scanner.

### 2.5 Generation of cortical depth streamlines

For each subject, the high-resolution T1-weighted anatomical volume was segmented into different tissue types using *FreeSurfer* (Khan et al., 2011; Dale et al., 1999; Bajaj et al., 2008). The two cortical surface meshes were then generated: the gray-white matter interface and the pial surface. We then constructed a self-reciprocal depth coordinate system in the space between the two surface meshes. The resulting cortical thickness normalized depth coordinates, *w*, were then used for depth averaging and mapping the gray-matter data to the surface mesh (Truong et al., 2020; Kim et al., 2017).

### 2.6 Functional data analysis

Each functional dataset was corrected for slice acquisition timing, and head motion effects using a robust expectation–maximization method (Nestares and Heeger, 2000). Additionally, fMRI time-series was corrected for receiver-coil inhomogeneity spatial variations. Correction was also applied for low-frequency temporal baseline drifts. Ultimately, the corrected datasets were spatially aligned together and the corresponding high-resolution T1-weighted anatomical volume using the inplane anatomy collected in each session.

For each session, the time-series data, each containing a total number of 80 HRF events, were averaged across the central layers of gray matter to lessen partial volume effects. This process was performed by depth-averaging the time-series data on the range of *w* = 0.2–0.8, and this average was mapped onto the gray-white interface vertices. We also removed any of the HRF events with excessive head motion (>2 mm per TR) from the depth-averaged data. We then spatially smoothed the head-motion censored time-series data with an 8-mm FWHM Gaussian kernel, and transformed them into the MNI-152 standard space using the curvature-based surface registration approach implemented in *FreeSurfer* (Bajaj et al., 2008; Fischl et al., 1999; Collins et al., 1994). For each vertex on the standard surface, we performed bootstrapping (500 resamples with replacement) across HRF events to quantify variability at each time point (Efron, 1987; Efron and Tibshirani, 1994). Each bootstrapped HRF time-series was upsampled at a 0.1-s interval using spline interpolation. The time-series was then baseline-corrected by subtracting the average of its first and last time points. Each HRF was characterized with six parameters (Figure 1C): (1) peak amplitude, (2) Time-to-peak (TTP), (3) undershoot amplitude, (4) time-to-undershoot (TTU), (5) FWHM, and (6) HFPF; defined as the ratio of the power in the upper half of the frequency range, 0.2–0.4 Hz, to the total power (Fesharaki et al., 2024). This model-free approach obtains HRF parameters directly from the fMRI data without the need for specific fitting to a pre-specified shape. The bootstrapped time series generated above were then used to evaluate the reliability of the model-free parameter estimates. We also calculated the peak-amplitude CNR for each HRF – defined as the ratio of the peak amplitude to its standard error of the mean. For each vertex, we computed the bootstrapped mean for each parameter. A linear mixed-effects model was then used to assess the main effects of age and sex, with session ID included as a random effect. Finally, brain regions showing statistically significant (*p* < 0.05) differences with age for each parameter were identified. To reduce spatial multiple-comparisons issues, we used a minimum cluster size threshold of 72 mm^2^, the equivalent area of our 8-mm FWHM smoothing kernel.

## 3 Results

Using the SAST, strong HRFs (CNR > 3) were evoked across the majority of the cerebral cortex. The fraction of cortex exhibiting CNR > 3 (cortical coverage) varied across subjects, ranging from 50.8% to 99.0%, with a mean of 87.8% ± 10.6%. Cortical coverage averaged 87.9% ± 10.07% (range: 50.8%–96.9%) in young individuals and 87.6% ± 11.3% (range: 59.4%–99.0%) in older adults. Consistent with our previous study (Fesharaki et al., 2024), aging did not significantly affect cortical coverage (Wilcoxon rank-sum test, *p* = 0.41).

### 3.1 Regional changes in HRF amplitudes across age

**Figure 2:**
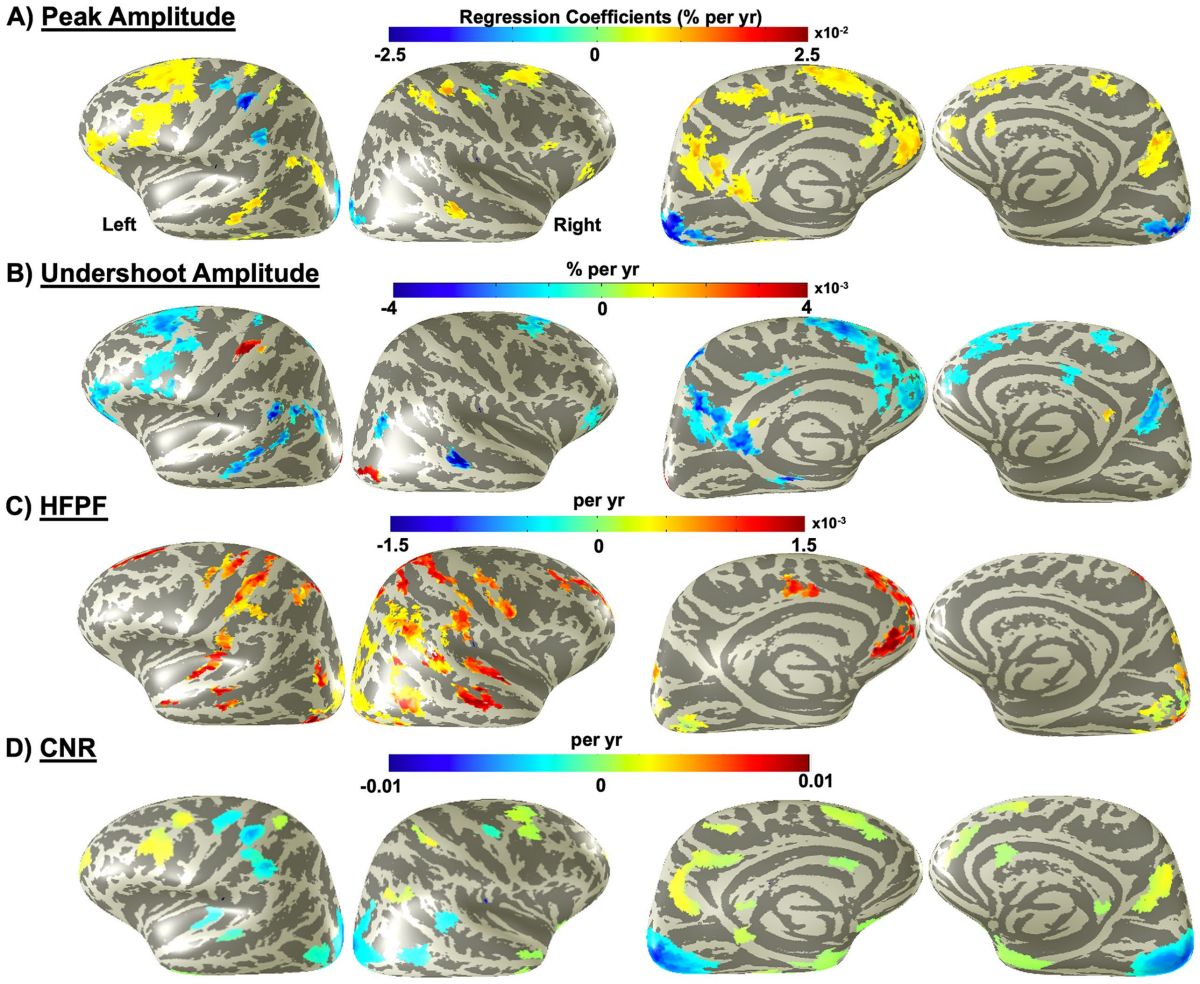
Distribution of brain regions with significant (*p* < 0.05) age-related changes across the cortex for HRF parameters: **(A)** Peak amplitude, **(B)** Undershoot amplitude, **(C)** High-frequency power fraction (HFPF), and **(D)** Contrast-to-noise ratio (CNR). Color overlays indicate linear regression slopes.

Large regions of cortex exhibited age-related variations that were significant (*p* < 0.05) across subjects (Figure 2). Regression slopes of peak amplitude generally indicated a gradual but significant increase with age, ranging from 0.005 to 0.01% per year (Figure 2A). Increases were primarily observed in the left frontal lobe, particularly, in motor and premotor areas, ventrolateral prefrontal cortex, and lateral frontopolar cortex, and to a lesser extent, in the right frontal lobe. Peak amplitude also showed significant increases in some medial areas of the parietal lobe, particularly in multisensory integration areas. Together, these regions accounted for 14.5% of overall brain activation (Table 1). However, a smaller portion of the brain (4.4%) showed decreases in peak amplitude with age. These negative changes were observed in several primary sensory areas, including somatosensory cortex – specifically in the left hand-knob area and its associated supplementary regions, as well as bilaterally in early visual cortex (Figure 2A, bluish areas).

**Table 1:** Proportions of the cortex with significant age-related changes in HRF amplitudes across age.

Parameters	Affected Cortex (%)	Positive-slope cortex (%)	Negative-slope cortex (%)	Positive-slope fraction (%)	Negative-slope fraction (%)
Peak amp.	18.9	14.5	4.4	76.6	23.4
Undershoot amp.	16.7	2.6	14.1	15.4	84.6
HFPF	19.3	18.9	0.4	98.2	1.8
CNR	19.8	10.4	9.4	57.5	47.5

Peak amp, peak amplitude; Undershoot amp, undershoot amplitude; HFPF, high-frequency power fraction; CNR, contrast-to-noise ratio.

Age-related undershoot amplitude generally mirrored the age-related effects observed in peak amplitude, although changes in regression slopes were smaller in magnitude, ranging mainly from −0.003% to −0.001% per year (Figure 2B), and were confined to a more limited portion of the cortex (16.7%; Table 1). Notably, some regions that showed age-related effects for peak amplitude, such as the early visual cortex bilaterally and the right lateral and left medial parietal lobes, did not exhibit similar effects for undershoot amplitude. Consequently, a smaller portion of the areas affected by age showed decreasing effects for undershoot amplitude compared to peak amplitude (Table 1).

For the HFPF, overlays indicated age-related increases, particularly in posterior regions of the right hemisphere laterally, frontal regions of the left hemisphere medially, bilaterally in early visual cortex, and in certain areas of the frontoparietal and temporal lobes (Figure 2C). Altogether, 19.3% of the cortex exhibited significant age-related changes in HFPF. Of these, only 1.8% showed decreases with age, primarily located in the early visual cortex (Figure 2C; Table 1).

CNR showed age-related effects largely overlapping with those observed for peak amplitude, though with more extensive involvement in regions where peak amplitude decreased with age, and to a lesser extent, in regions where it increased. Although the overall proportions of affected cortex were similar for the two parameters (18.9% for peak amplitude vs. 19.8% for CNR), a greater proportion of cortex exhibited decreases in CNR with age (9.4% vs. 4.4%). This discrepancy was partly driven by additional regions where CNR decreased with age, including bilateral areas of the temporal lobe, specifically, anterior medial and posterior lateral regions (Figure 2D).

### 3.2 Regional changes in HRF timing parameters across age

**Figure 3:**
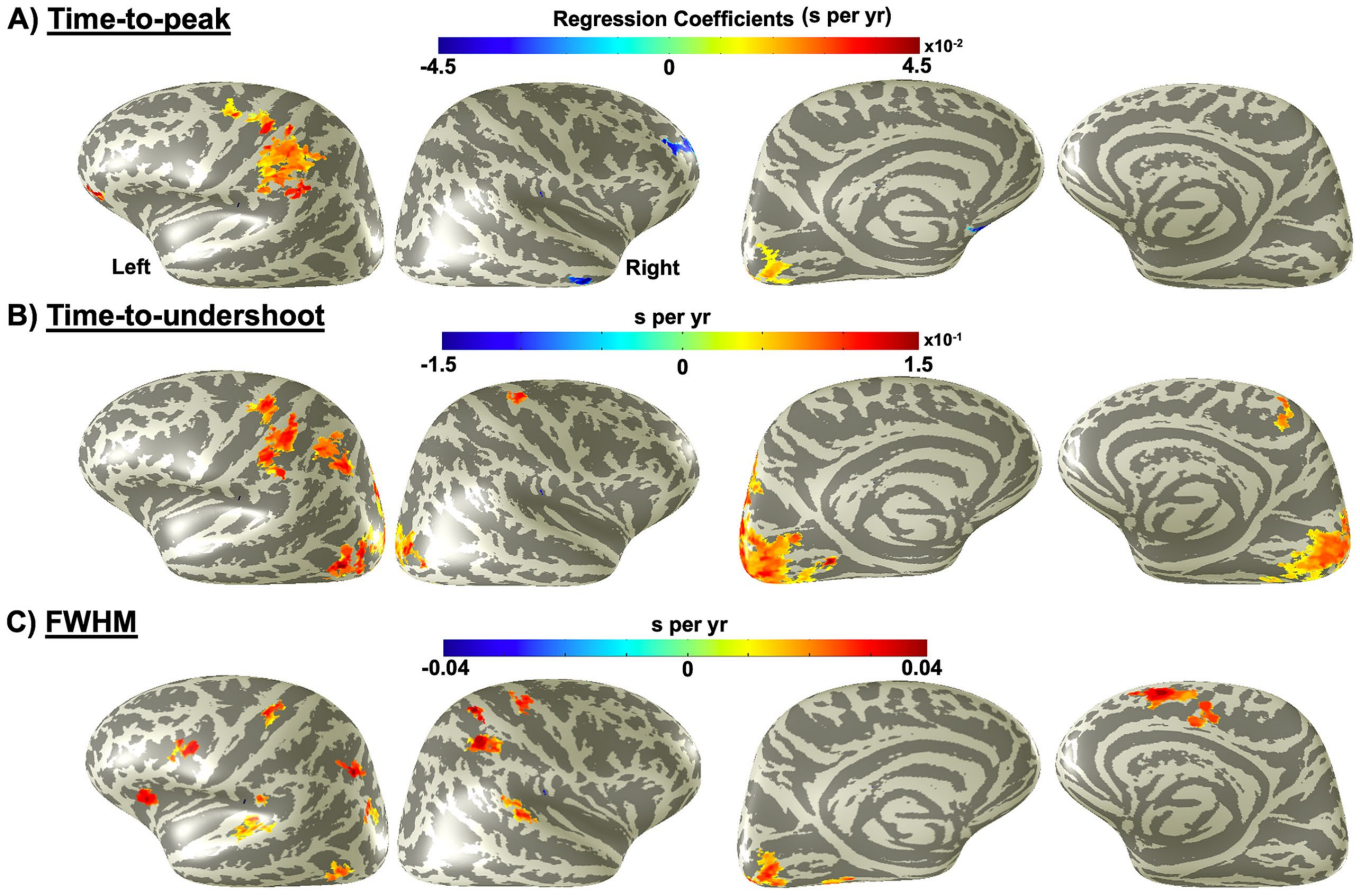
Distribution of brain regions with significant (*p* < 0.05) age-related changes across the cortex for the hemodynamic response function’s **(A)** time-to-peak, **(B)** time-to-undershoot, and **(C)** full width at half maximum.

Compared to the age-related effects observed in amplitude parameters, changes in the temporal parameters of HRFs with age were evident in far fewer cortical areas (Figure 3). On average, only 9.4, 13.6, and 10.2% of cortex showed significant age-related changes in TTP, TTU, and FWHM, respectively (Table 2).

**Table 2:** Proportions of the cortex with significant age-related changes in HRF timing parameters across age.

Parameters	Affected cortex (%)	Positive-slope cortex (%)	Negative-slope cortex (%)	Positive-slope portion (%)	Negative-slope portion (%)
TTP	9.4	6.9	2.5	73	27
TTU	13.6	12.2	1.4	90	10
FWHM	10.2	8.6	1.6	83.9	16.1

TTP, time-to-peak; TTU, time-to-undershoot; FWHM, full-width at half maximum.

In general, certain cortical regions exhibited age-related increases in both TTP and TTU, indicating slowing dynamics with age (Figure 3A,B). For TTP, this effect was particularly prominent in the left lateral parietal lobe, and, to a lesser extent, the left early visual cortex. However, a very small portion of the brain (e.g., the right lateral prefrontal cortex) showed significant age-related decreases in TTP with age (2.5; Table 2).

Compared to TTP, a larger proportion of the cortex showed significant age-related increases in TTU (13.6%; Table 2), with more extensive bilateral involvement in the early visual cortex. Delayed undershoot timing was also observed in the left lateral parietal lobe, though to slightly a lesser extent, with some extension into the parieto-occipital region (Figure 3B). However, the age-related changes in TTU were smaller in magnitude, ranging from 0.05 to 0.15 s per year, with approximately 90% of the affected areas showing a slower TTU (Figure 3B; Table 2).

A few small, spatially localized regions showed significant age-related changes in FWHM (Figure 3C). These regions primarily exhibited positive slopes (8.6% of the total 10.2% affected areas; Table 2), with increases ranging from 0.005 to 0.04 s per year, indicating slower dynamics with age. For example, increases (0.01 to 0.03 s per year) were observed in left early visual cortex, nearly matching the extent seen for TTP, as well as ventral occipital cortex. This effect was also evident in several scattered clusters, including both lateral and medial areas of the right parieto-frontal cortex, as well as the left frontal cortex, ventral prefrontal cortex, and parieto-occipital regions. Additionally, significant increases were observed in a small region of the lateral temporal lobe.

## 4 Discussion

In our previous work, we examined age-related trends in the dynamics of the HRF globally across cerebral cortex within each subject’s native space. Those findings provided evidence of systematic alterations in NVC with age, consistent with established patterns of microvascular aging (Xu et al., 2017; Tarumi and Zhang, 2018). Building on this foundation, the present study investigated region-specific age-related changes in HRF amplitude and timing parameters, using sex-balanced groups of younger (22–30 years) and older (54–75 years) adults in a standard space. We hypothesized that regional variations in HRF characteristics would follow spatial patterns consistent with vascular aging that were consistent with the global results.

Our results showed that distinct regions of the cortex exhibit substantial age-related effects in both amplitude and timing parameters of the HRF. However, age-related changes in HRF amplitudes were observed across a noticeably larger proportion of the cortex – nearly twice as much – compared with changes in HRF timing parameters (Tables 1 vs. 2). Although the effects of aging for HRF parameters differed somewhat across cortical regions, their spatial patterns displayed partial overlap. For example, TTP and TTU exhibited broadly similar regional distributions, as did peak amplitude, undershoot amplitude, and CNR – consistent with prior work demonstrating correlations among these parameters (Zong and Huang, 2011; Hu et al., 1997; Taylor et al., 2018).

Spatial patterns in peak amplitude changes indicated that aging was associated with increased responses in most regions, with 77% demonstrating elevated peak amplitudes (Table 1). Increases were most prominent in the frontal lobe, predominantly within the left hemisphere (Figure 2A). Notably, these effects were concentrated in the lateral posterior regions, encompassing areas associated with the premotor cortex, as well as the orbitofrontal and ventrolateral prefrontal cortices. Elevated peak amplitudes were also observed bilaterally in the medial parieto-occipital regions and, to a lesser extent, in the medial parietal and lateral temporal lobes – once again, more extensively in the left hemisphere (Figure 2A). The left-lateralized pattern may be driven by the task design, which required a right-handed motor response (Figure 1), or it may reflect an actual age-dependent vascular asymmetry (see below).

Taken together, these spatial distributions suggest that brain regions involved in multisensory integration, information processing, and higher-order cognitive functions are more susceptible to age-related increases in peak amplitude – and, to a lesser extent, in undershoot amplitude (Figure 2A,B). This pattern aligns with studies reporting multisensory enhancement in older adults, thought to reflect compensatory mechanisms for age-related declines in peripheral sensory processing or increased baseline cross-modal interactions associated with aging (Mozolic et al., 2008; Laurienti et al., 2006; Peiffer et al., 2007; Diederich et al., 2008).

By contrast, age-related decreases in peak amplitude were observed in a smaller subset of regions (23%, Table 1), primarily within areas involved in sensorimotor and visual processing (Figure 2A). This finding diverges from several earlier studies that reported no significant age-related changes in these regions (D’Esposito et al., 1999; Buckner et al., 2000; Huettel et al., 2001; Aizenstein et al., 2004; Asemani et al., 2017; Morsheddost et al., 2015). Nevertheless, other studies have documented significant reductions in HRF amplitude with age, particularly within the visual cortex (Buckner et al., 2000; Morsheddost et al., 2015; Ross et al., 1997). One likely explanation for these discrepancies lies in methodological differences, including the use of a canonical HRF in deconvolution and/or reliance on group-averaged ROI analyses, both of which may obscure individual variability or regional specificity. More recently, McDonough et al. (2025) estimated subject-specific HRFs from voxels exhibiting maximal responses during a flashing checkerboard task in occipital cortex and similarly reported age-related reductions in HRF peak amplitude (McDonough et al., 2025).

The spatial patterns and trends of age-related effects in undershoot amplitude broadly mirrored those observed for peak amplitude but were smaller in magnitude and more spatially restricted (Figure 2A,B; Table 1). This correspondence is consistent with previous findings (Zong and Huang, 2011; Hu et al., 1997; Taylor et al., 2018), and may reflect the generally weaker magnitude of the undershoot relative to the peak response.

Notably, although peak amplitude showed age-related reductions in early visual cortex, undershoot amplitude did not. This observation extends prior findings that have suggested a strong correspondence between these parameters (Zong and Huang, 2011; Taylor et al., 2018). It should be noted, however, that those studies did not include older adults, making it unclear whether this relationship would hold across a broader age span. Another possibility is that the pronounced reduction in peak amplitude observed in early visual cortex reflects neuronal rather than vascular changes (Handwerker et al., 2007). Alternatively, the reduction in peak amplitude may have simply made the corresponding undershoot too weak to discern age-related changes.

For peak amplitude, our previous work did not reveal any significant global age-related changes in spatial mean (Fesharaki et al., 2024). One possible explanation is that averaging across the cortex may obscure regional differences. This interpretation is consistent with our earlier observations of global age-related declines in the spatial variability of peak amplitudes.

Our results also revealed age-related reductions in CNR across the visual and sensorimotor cortices, with broader spatial extent but smaller magnitude than the changes observed for peak amplitude (Figures 1D vs. 1A). This pattern suggests elevated variability in regions particularly vulnerable to age-related degradation, specifically within the territories of the MCA and PCA (Roth et al., 2017; Flück et al., 2014). Reductions in CNR were also evident in small regions of the temporal lobe without corresponding decreases in amplitude, further indicating increased variability with aging (Supplementary Figure S1). Conversely, a few small regions showing age-related increases in CNR overlapped with areas exhibiting increased peak amplitude; however, these CNR increases were modest compared with amplitude changes, again suggesting increased variability (Supplementary Figure S1).

These findings align with previous reports that aging is generally associated with increased noise in the BOLD signal, driven primarily by physiological and vascular factors (Lu et al., 2011; Tsvetanov et al., 2015; Garrett et al., 2010; Grady and Garrett, 2014). Previously, we reported global age-related declines in both the spatial mean and standard deviation of CNR (Fesharaki et al., 2024). The current findings, however, indicate that these effects vary regionally, with some showing increased CNR and others showing decreased CNR, consistent with prior work that documented region-specific heterogeneity in signal variability with aging (Grady and Garrett, 2014). Once again, this discrepancy may have arisen from averaging across cortex in global analyses, which can obscure regional differences, so that only the dominant age-related effect of decreased CNR prevailed.

Next, we evaluated the effects of aging on the temporal characteristics of the HRF across cortical regions. Our results showed that aging also influences the HRF timing, with distinct regional patterns. Specifically, we observed age-related increases in both TTP and TTU, indicating slower temporal dynamics of the HRF with age in a region-specific manner. However, the spatial extent of delayed undershoot was greater than that of delayed peak (13.6% vs. 9.4%; Table 1). This extent difference was most prominent bilaterally in early visual cortex (Figures 3A,B). Additionally, although the rate of delay was larger for undershoot than for peak, with TTU and TTP increasing by 0.05–0.1 and 0.01–0.04 s per year, respectively (Figures 3A,B), the relative changes in TTP and TTU showed less disagreement, which reduced the overall magnitude of this effect.

In early visual cortex, we observed age-related declines in peak amplitude alongside delayed undershoot amplitude. One possible explanation is that feedforward neural signaling to local blood vessels, which triggers vasodilation in response to neural activity, may remain relatively preserved with age, whereas the return of CBF to baseline becomes progressively slower. This interpretation aligns with previous studies reporting more prominent age-related effects on HRF offset than onset timing (Lu et al., 2011; Tsvetanov et al., 2015; D’Esposito et al., 2003). Age-related increases in both TTP and TTU were also consistently observed in the left lateral parietal lobe (Figures 3A,B), suggesting a shared aging trajectory in this region. Importantly, however, the age-related changes in TTP and TTU observed in both early visual cortex and lateral parietal lobe likely reflect alterations in vascular and hemodynamic processes rather than differences in neural activity (Handwerker et al., 2007).

Notably, lateral parietal cortex is primarily perfused by MCA, whereas early visual cortex is supplied by PCA (Rey et al., 2010). This anatomical distinction suggests that regional differences in HRF timing may arise from the differential vulnerability of major cerebral arteries to vascular aging. Supporting this interpretation, reduced PCA flow responses to visual stimulation have been reported in older populations, most likely as a result of disrupted NVC (Panczel et al., 1999; Zheng et al., 2003; Niehaus et al., 2001). Furthermore, age-related stiffening of large arteries (e.g., ACA, MCA, and PCA) impairs CBF regulation and transmits pressure pulsations to downstream vessels, which ultimately contribute to microvascular damage and reduced vascular compliance associated with hypertension (Jennings et al., 2021; Björnfot et al., 2021; Wang et al., 2018; Harvey et al., 2015). Consistent with these aging affects, transcranial Doppler ultrasound studies have demonstrated age-related reductions in both resting CBF and stimulus-evoked CBF responses, likely associated with increased arterial stiffness within both MCA and PCA territories (Flück et al., 2014; Müller and Schimrigk, 1994; Demirkaya et al., 2008). However, some studies report a greater age-related decline in resting CBF for the MCA than the PCA (Flück et al., 2014; Müller and Schimrigk, 1994). Complementary evidence from 4D flow MRI studies quantifying cerebral arterial stiffness have revealed age-related increases in pulse wave velocity, particularly within the ACA territory, further supporting the age-related stiffening of large arteries (Björnfot et al., 2021). Nevertheless, age-related pathological differences between posterior and anterior large arteries have also been documented, including more pronounced concentric intimal thickening, increased wall thickness, and reduced arterial elasticity in vessels of the posterior cerebral circulation (Roth et al., 2017). Moreover, despite an overall age-related reduction in vessel density across territories supplied by large arteries, magnetic resonance angiography studies have demonstrated the greatest vessel loss within the PCA circulation, and to a lesser extent, within the left MCA circulation (Xu et al., 2017; Bullitt et al., 2010). Together, these findings suggest that the PCA may be more vulnerable to advanced cerebrovascular aging relative to the ACA. Our findings of greater age-related effects in posterior occipital lobe also support this observation. Altogether, these studies, together with our results, support the conclusion that cerebrovascular aging is most distinct within the territories of the major cerebral arteries.

The current results also reveal notable asymmetries: significant age-related changes in both TTP and TTU were unilateral in the parietal lobe, whereas in early visual cortex, TTU changes were bilateral and TTP changes unilateral (Figures 3A,B). These asymmetries could be related to observed asymmetries in cerebrovascular pathology, such as the greater reduction in vessel density reported in the left compared with the right MCA territory, as discussed above (Xu et al., 2017; Bullitt et al., 2010). Unilateral impairment of the MCA has been associated with established cerebrovascular pathologies such as ischemic stroke, which may exert greater effects in older adults due to pre-existing vascular changes (Jüttler et al., 2014; Alwatban et al., 2021). Consistent with our findings, there is also evidence that left-hemisphere MCA stroke occurs more frequently in older adults (Gottesman et al. 2008). Therefore, the observed asymmetries in TTU and TTP may reflect underlying anatomical or vascular asymmetries across hemispheres – potentially related to latent or developing cerebrovascular pathology.

Regarding FWHM, our previous work demonstrated age-related increases in the global spatial standard deviation of FWHM, and interesting changes in its spatial distribution (Fesharaki et al., 2024). In contrast, the region-specific analyses in the present study revealed only small and scattered clusters of significant FWHM changes. These results align with prior research demonstrating a general trend toward slower hemodynamic responses with advancing age (West et al., 2019; Henson et al., 2024). The observation that the large global effects mostly disappeared when averaged across subjects in a standard space is consistent with patchy, small-scale age-related changes that mostly do not survive averaging. It is possible that the effects that remain are those situated near the watershed territories of the large cerebral arteries, where age-related vascular pathology is most likely to occur (Momjian-Mayor and Baron, 2005; Sorond and Lipsitz, 2011).

Our finding that aging was associated with delayed HRF and an increase in width of HRF within visual cortex differs from the results reported by McDonough et al. (2025) (McDonough et al., 2025). In their study, a flashing checkerboard task was used to estimate subject-specific HRFs from voxels showing maximal responses in occipital cortex. However, deriving a single subject-specific HRF per individual may obscure regional variability in HRF dynamics, which we quantified extensively in previous work (Taylor et al., 2018). An additional explanation may relate to task-dependent sensitivity, as voxels within visual cortex may respond differently to a flashing checkerboard task than to the SAST. Consequently, estimating subject-specific HRFs at the voxel level across the cortex may yield findings more consistent with those observed in the present study.

Moreover, we previously demonstrated a global increase in the variability of HRF temporal dynamics with age (Fesharaki et al., 2024). However, the present findings indicate that such variability is attenuated in regions with stronger and more spatially reliable perfusion – particularly, in the lateral parietal and early visual cortices. Consistent with physiological evidence (Bennett et al., 2024; Tsvetanov et al., 2021), this suggests that age-related disruptions in blood delivery through the large cerebral arteries may emerge gradually and in a spatially heterogeneous (i.e., patchy) manner. Consequently, when averaged across individuals, age-related changes in HRF temporal dynamics tend to manifest only in localized cortical regions close to the territories of the ACA, MCA, and PCA.

Regarding HFPF, our previous work demonstrated that both its global mean and standard deviation dramatically increase with age (Fesharaki et al., 2024). In the present study, unlike FWHM, HFPF changes were clearly evident across large portions of cortex when averaged across individuals (Figure 2C), consistent with our global findings. This widespread pattern also indicates greater spatial regularity of these effects across subjects. Importantly, such widespread yet regionally specific patterns of significant age-related effects are unlikely to arise from rigid–body head motion, which would be expected to introduce more global or spatially edge-dominated artifacts. Moreover, while HFPF reflects higher–frequency components of the HRF and is intended to characterize the fast HRF dynamics, head motion typically occurs slowly, and its effects predominantly manifest as low-frequency signal fluctuations rather than sustained high-frequency components. These considerations suggest that the residual head motion is unlikely to be the primary driver of the observed age-related increases in HFPF. Additionally, the spatial distribution of HFPF regression slopes indicated that age-related HFPF changes may follow cortical curvature, which anchors the transformations from native-to-MNI space. Given that cerebral vascular architecture closely follows gyral and sulcal geometry (Duvernoy et al., 1981), we therefore examined whether age-related HFPF increases differ between gyri and sulci (Supplementary Figure S2). Indeed, using cortical curvature to separate gyral and sulcal contributions, we observed a statistically significant trend (*p* < 0.0001), indicating greater age-related HFPF increases in gyri compared with sulci (Supplementary Figure S2). Gyri lie closer to pial arteries and therefore experience less damping of arterial pulsations, which allows stronger propagation of high-frequency pressure waves. With aging, arterial stiffening amplifies this pulsatile effect, particularly in gyral regions (Lin et al., 2021). Although significant, this effect is noisy, likely due to averaging across brains with idiosyncratic geometries. Certainly, further work is justified to fully elucidate the mechanisms linking cortical geometry, vascular aging, and HRF high-frequency dynamics.

Our study has several limitations. First, our region-based analysis was restricted to brain areas activated by the SAST. Because these regions can vary somewhat across subjects (Fesharaki et al., 2024; Taylor et al., 2018), this variability introduces a potential confound, and limits the spatial scope of the data. Second, although the SAST robustly activates the majority of the cerebral cortex (about 87% on average), it evokes weaker activation in some brain regions, such as limbic area and parts of the prefrontal cortex. Consequently, the age–related effects observed in these regions could be less reliable. Future studies employing tasks that more strongly engage these regions are therefore needed to validate the robustness of the age-related findings in these brain regions. Third, participants performed a low-demanding color-detection fixation task during the inter-stimulus intervals to prevent attentional drift. However, this task may itself alter brain state and introduce minor confounds to the HRF dynamics (Li et al., 2016). Fourth, although participants were classified into two discrete age groups of young adults (22–30 years) and older adults (53.5–75 years) to achieve a balanced sex distribution within each group, this grouping may obscure continuous age-related trends in HRF changes, particularly across middle age (30–50 years). Additionally, our results may be affected by false positives and false negatives *simply due to our modest sample size*. Therefore, future studies with larger sample size and more continuous age sampling between younger and older adults are warranted to address this issue. Fifth, our region-based analysis was carried out in standard space. Therefore, future work should examine the affected areas in each individual’s native space, potentially incorporating additional measurements such as blood flow and oxygen metabolism to better disentangle age-related effects inherent to the BOLD signal. Sixth, in the present study, there was no systematic assessment of cerebrovascular and metabolic factors, such as blood pressure, lipid profiles, smoking history, and diabetes. While these factors are known to influence NVC and may therefore affect HRF measurements, future studies incorporating comprehensive cerebrovascular and metabolic profiling will be crucial for better interpretation of age-related changes in HRF dynamics. Finally, although we controlled for sex in our age groups and observed notable sex differences, further validation and detailed characterization of these effects are required and will be addressed in a subsequent study.

In conclusion, comparing spatially resolved cortical HRF dynamics between sex-balanced groups of young and older adults in standard space demonstrated widespread yet spatially heterogeneous changes in both amplitude and timing characteristics across cortex. The region-specific analyses identified distinct age-related alterations, particularly within territories of the major cerebral arteries and their watershed areas. For amplitude parameters, aging was associated with declines in visual and sensorimotor areas and increases in regions involved in higher-order cognitive and decision-making processes. Aging also produced delayed HRF timing, with the most prominent effects in early visual and lateral parietal cortices. These regional patterns are consistent with known age-related vascular changes, including increased arterial stiffness, greater vulnerability of posterior cerebral circulation, and reduced cerebrovascular responsiveness. Moreover, these findings align with our previous work demonstrating global effects of aging on the HRF, particularly with respect to HFPF. However, direct comparison between the present spatially localized results – derived from sex-balanced groups and analyzed in the standard space – and our earlier global analyses revealed important differences. In particular, we observed extensive age-related effects on FWHM at the global level that were not apparent in the spatially resolved analyses, suggesting that the globally evident FWHM changes reflect either spatially fine-grained characteristics or idiosyncratic subject-to-subject difference that do not survive transformation and analysis in the standard space. In contrast, peak amplitude and time-to-peak exhibited clear region-specific age-related alterations that were not evident in native-space mean measures or their variability. From a methodological perspective, these findings are a warning that purely standard-space cortical analyses can obscure meaningful experimental results. Both native-space and standard-space evaluations are needed for thorough understanding.

Taken together, results from both global and spatially resolved analyses indicate that aging profoundly affects NVC and underscore the importance of accounting for vascular aging when interpreting BOLD signals in older adults. Future studies are required to further elucidate and quantify the underlying mechanisms that give rise to HRF changes across the lifespan.

## Data Availability

The raw data supporting the conclusions of this article will be made available by the authors, without undue reservation.
