# Supplementary material for: Age-related variations of the hemodynamic response function spatially resolved across human cerebral cortex
Source: Front Aging Neurosci. 2026 Feb 25;18:1774543. doi: 10.3389/fnagi.2026.1774543 (PMC12975896; doi:10.3389/fnagi.2026.1774543)
Supplement: Supplementary file 1 [file Table_1.docx]

Age-related variations of the hemodynamic response function spatially resolved across human cerebral cortex

Nooshin J. Fesharaki^1^, Amanda Taylor^2^, & David Ress^3*^

^1^Neurosurgery Department, University of Texas Health Science Center at Houston, TX, USA

^2^Physics and Engineering Department, University of St. Thomas, Houston, TX, USA

^3^Department of Neuroscience, Baylor College of Medicine, Houston, TX, USA

**Supplementary materials**

**Age-related changes in SEM**

For each vertex, we computed the standard error of the mean (SEM) – defined as the ratio of the bootstrapped mean of the peak amplitude to its contrast-to-noise ratio. A linear mixed-effects model was used to assess the main effects of age and sex, with session ID included as a random effect. Brain regions showing statistically significant (*p* < 0.05) differences with age were then identified (Fig. S1).


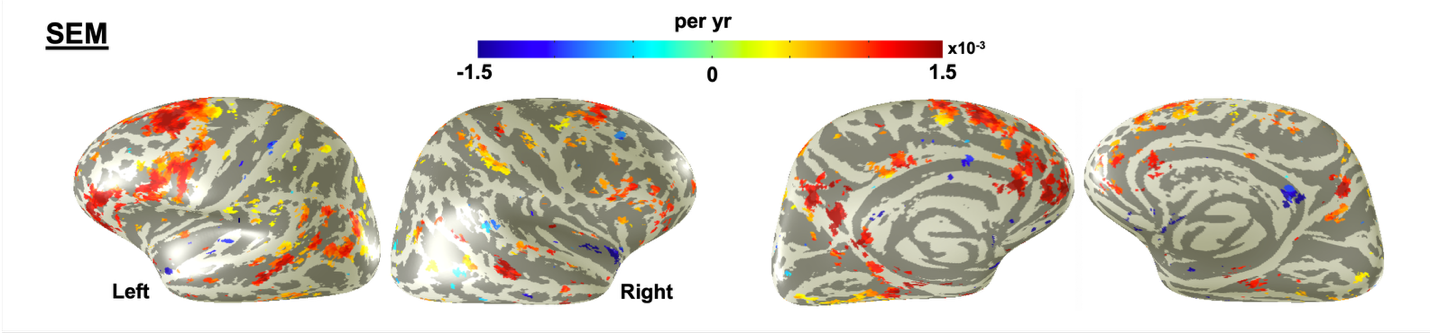


**Figure S1.** Distribution of brain regions with significant (*p* <0.05) age-related changes across the cortex for the HRF peak amplitude’s standard error of the means (SEM). The color bar indicates linear regression slopes.

**Relationship between age-related changes in HFPF and cortical curvature**

For cortical regions showing statistically significant (*p* < 0.05) age-related changes in the high-frequency power faction (HFPF), we examined whether there was a correlation between their spatial distribution and cortical geometry. At each voxel, we therefore estimated the Gaussian curvature of the *FreeSurfer* cortical surface mesh as curvature $= \frac{D}{\left| D \right|+\sqrt{A} /4}$ , where *D* is the signed distance for the vertex with respect to the best-fit plane containing its connected neighbors, and A is the barycentric area of the surrounding triangles featuring that vertex. Therefore, positive curvature corresponds to gyri (convex regions), negative curvature corresponds to sulci (concave regions), and zero curvature refers to a locally flat surface.

Our results showed a statistically significant but noisy trend (*p* < 0.0001) for age-related increased HFPF in gyral regions compared with that in sulcal regions (Fig. S2). This variability of this effect likely results from averaging across brains with idiosyncratic cortical geometries.


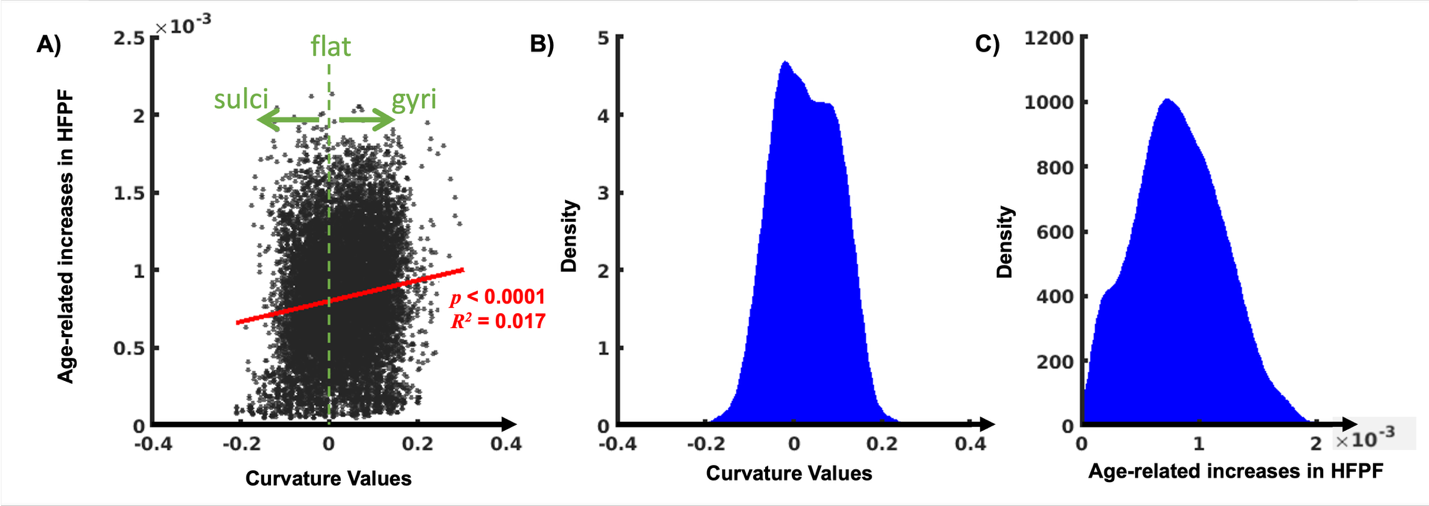


**Figure S2.** (**A**) The scatter plot shows the distribution of high-frequency power fraction (HFPF) changes with age within the sulci and gyri. There is a significant (*p* <0.0001) trend towards positive curvatures (gyri). The red solid line is the linear regression line. *R* = Pearson correlation coefficient. (**B**) and (**C**) show the probability density distributions for curvature values and age-related changes in HFPF, respectively.
